# Natural language processing for electronic health records in anaesthesiology: an introduction to clinicians with recommendations and pitfalls

**DOI:** 10.1007/s10877-024-01128-3

**Published:** 2024-02-04

**Authors:** Martin Bernstorff, Simon Tilma Vistisen, Kenneth C. Enevoldsen

**Affiliations:** 1 Department of Affective Disorders, Aarhus University Hospital – Psychiatry, Aarhus, Denmark; 2 Department of Clinical Medicine, Aarhus University, Aarhus, Denmark; 3 Center for Humanities Computing, Aarhus University, Jens Chr. Skous Vej 4, Aarhus N, 8200 Denmark; 4 Department of Anaesthesiology and Intensive Care, Aarhus University Hospital, Palle Juul-Jensens Boulevard 99, Aarhus N, 8200 Denmark; 5 Quantitative Genomics Group, Aarhus University, Aarhus N, Denmark

**Keywords:** Natural language processing, Large language model, Machine learning, Prediction model, Surgical duration

The application of advanced statistical models has become omnipresent in submissions to medical journals. Great results have been achieved within e.g. imaging segmentation and diagnostics [1], whereas other areas such as prediction models based on Electronic Health Records (EHRs) are still in their infancy when it comes to demonstrating added value [2].

Recently, large language models (LLMs) have reached a utility inflection point. LLMs are trained on text data, and studies where clinical *text* data is leveraged by advanced statistical modelling have not yet been published extensively in the *Journal*. Zhong and colleagues, a transdisciplinary author group, recently published a study that aimed to predict case duration of wrist fracture surgery, using the descriptive text from wrist X-ray radiology reports along with other hospital-, surgeon-specific, and demographic variables available prior to surgery. Their manuscript serves as a great example of how text data may find its utility within the anaesthesia field, and it seems likely that text data is soon going to get a more prominent place in the medical literature. In this editorial, we give a brief introduction to text data analyses.

## Representing text as numbers

Clinical notes often include information not present within structured EHR data and can contain important information for tasks such as predicting surgical case duration, mechanical restraint of psychiatric patients, etc. [3, 4]. To include this information within a model (e.g. logistic regression), the text needs to be represented as vectors. To do so, the text is often split into segments, called **tokens**, which, e.g. could represent words (“Rotwein”; German for red wine), subwords (“rot-wein”), or noun phrases (“red wine”). The concept, *token*, can be thought of as a generalization of the concept of *word* and allows a unit of representation which generalizes across languages.

Representing a sequence of tokens as a vector is often referred to as an **embedding**.

**Fig. 1:**
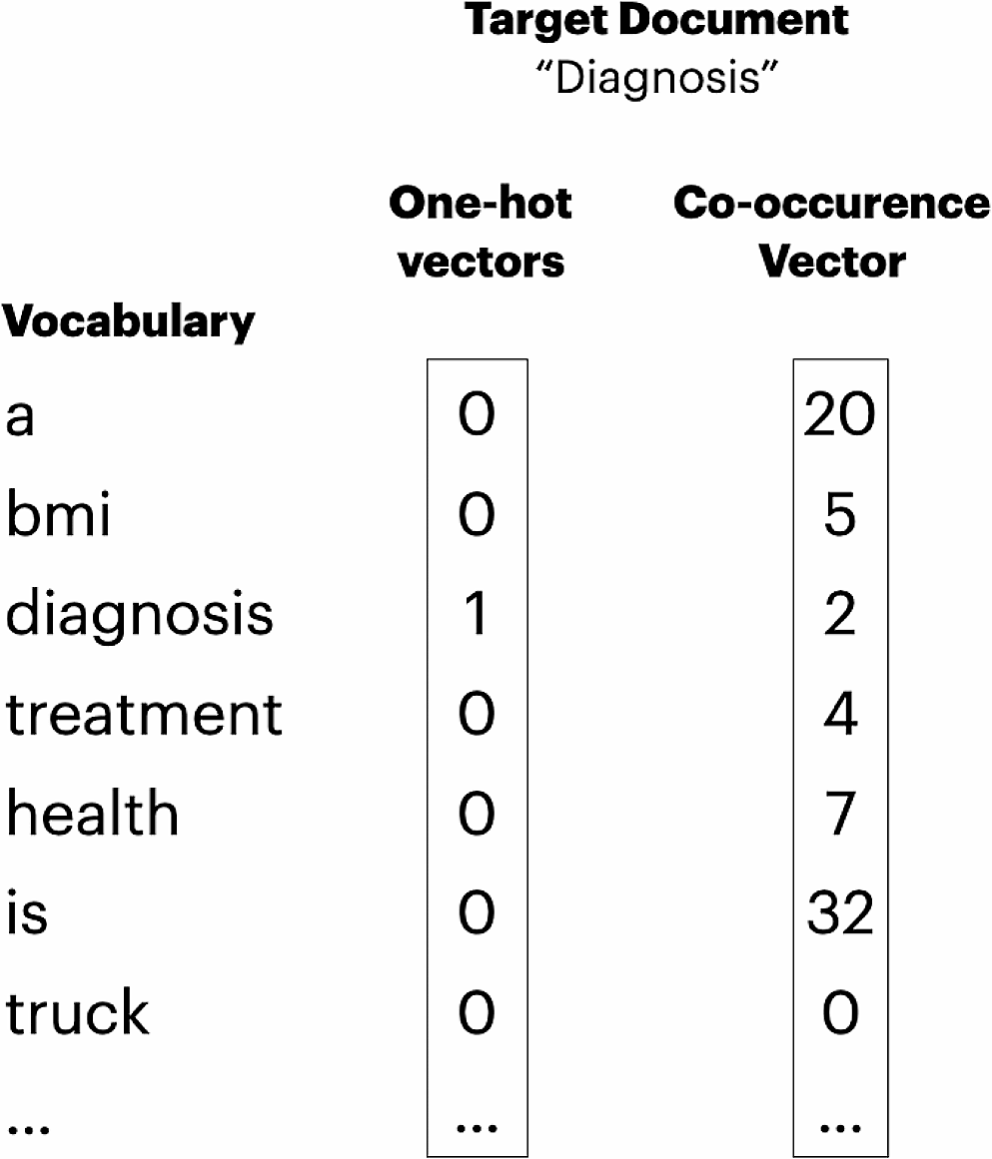
Showing two types of *(static)* vector representations, based on a one-hot encoding and a co-occurrence encoding, respectively

Tokens can be represented in various ways; one of the simplest is the one-hot encoding. In this method, each token in a vocabulary is given a unique number and represented by a vector of zeros, with a ‘one’ at its specific number’s position (see Fig. 1). However, this method has limitations, as it doesn’t indicate if two words are similar. This means that e.g. a spelling error (“mandible”/“mandble”) or synonyms (“mandible/lower jaw”) lead to entirely different one-hot encodings.

This is solved by representations based on the co-occurrence patterns in which a word appears [5, 6]. Consider the incomplete sentence:

Antidepressants are a class of medications used to ___ […].

Source: Wikipedia, Antidepressant, 2. Oct. 2023.

The reader might fill in words like “treat” or “prevent”. Words like these – which take on similar roles in a context – frequently co-occur with a subset of other words. Numerically, each word is represented by how often it co-occurs with another word from the vocabulary (see representation in Fig. 1). These co-occurrences are estimated over a large corpus of text. It is common to be able to predict the token/word’s semantic attributes from its co-occurence vector, e.g. whether it is a verb/noun or whether a word is a diagnosis [6].

**Fig. 2:**
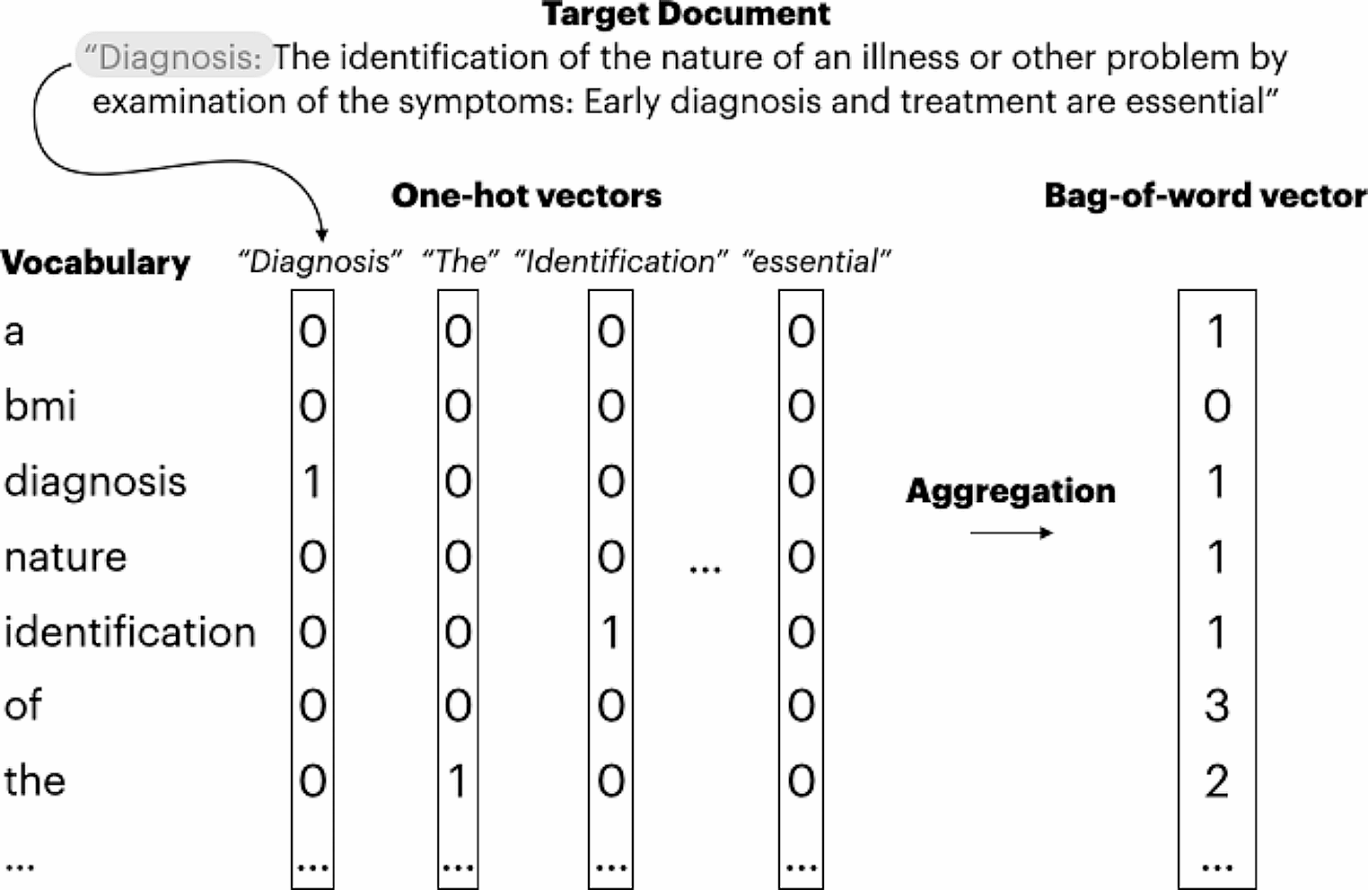
An example of aggregating word embeddings – in this case, summing one-hot representations creates a document representation using the counts of each word (bag-of-words)

However, in cases like the study of Zhong and colleagues, it is necessary to represent entire documents, not just individual words/tokens. One foundational approach is aggregating word embeddings. For instance, by summing one-hot-encoded word vectors, a so-called “bag-of-words” representation is derived, see Fig. 2. In this representation, each entry in the vector indicates the frequency of a specific word within the document.

There are more advanced methods available for capturing the intricacies of document structure and meaning. Techniques like recurrent neural networks (RNNs) [7] and transformers [8] can capture the contextual information of a document, which a simple aggregation might miss.

As demonstrated by Zhong and colleagues, these document embeddings can be integrated with structured data to create a *patient representation*. This representation can then be used for making predictions.

A fundamental limitation of the word vectors is their static nature, often referred to as “static word embeddings”. To illustrate, the vector representation for a word like “cell” remains unchanged regardless of its context, meaning it has the same vector whether referencing a “battery cell” or a “blood cell.”

Recognizing this limitation, there has been a shift towards contextual word vectors, where the embedding of a word is based on its context. Prominent LLMs such as BERT [5] and GPT [9] have pioneered this approach underpinned by recurrent neural networks and transformer methodology, which allows the modelling of context in a text to be less dependent on a single words’ position within that text. This is particularly relevant, because pronouns such as “it” or “they” are synonymous with words occurring several words back in a text, possibly in a previous sentence. LLMs generate contextualized word representations by predicting missing words in each sentence, mirroring the earlier example. Essentially, they leverage the surrounding context of a word to estimate its most appropriate representation. As an example, the previous sentence’s word “they” referred to “LLMs”, occurring 16 words prior in another sentence. LLMs can model this. Moreover, because they are heavily influenced by the data they’re trained on, domain-specific variants have emerged. For instance, ClinicalBERT [10], as highlighted by Zhong and colleagues, is tailored to capture the unique (English) linguistic patterns of the medical domain, where a term such as “cell” would seldom relate to batteries.

Using text for machine learning introduces more decisions into the analyses. This makes comprehensive documentation challenging and time-consuming. However, the choices are directly and unambiguously documented by the analysis code. When researchers share code, which Zhong and colleagues are commended for, they not only allow others to reproduce their methods, they also document their work and provide further explanations and clarifications of ambiguities.

For example, in the current study by Zhong and colleagues, the model also used historical scheduled skin minutes as a feature. This was not documented in the initial draft but was apparent from the shared code, available to reviewers. Adding this to the manuscript provides a better understanding of the model and the requirements for potential replication.

## Clinical pitfalls

It is a common trope in popular culture that knowing the future does not necessarily mean that it can be changed. This also applies to prediction models; it is important to focus development efforts on the modifiable endpoints. Which decisions are you trying to affect? How will they be different? Will the stakeholders act on a prediction presented to them?

This point may seem trite, but it is essential to justify the choice of analyses and to frame the potential pitfalls that follow. In the present study, the authors are aware of these possible limitations.

## Pointing out the obvious

Clinical notes contain both information pertaining to the patient (observations, measurements, and symptoms), and the healthcare workers’ reflections. For example, doctors frequently note down differential diagnoses, as well as the evidence for and against each diagnosis.

If a text model recognizes these differential diagnoses and uses them verbatim for diagnosis prediction, it might perform quite well on evaluation metrics, while it is of little use to the doctor, who had already considered a differential diagnosis. On the other hand, it may be incredibly useful to doctors who see the patient for the first time in the future. In psychiatry, for example, patient records of a thousand pages or more are not a rarity. If the pertinent information the model is communicating is found several years and 500 pages prior to the current consultation, the model can still be helpful.

Detecting such failure to create added value is tricky during model training. When the model is implemented, users will definitely notice if the model is merely parroting back what they already know.

Zhong and colleagues correctly compare the prediction models with a transparent reference model. Ideally, models should be presented alongside the problem they are intended to solve. If, for example, the model is designed to help with more time-efficient scheduling in surgery, it must outperform current practice to add value. Still, current practice may not be available in a dataset, so heuristics are often used in proof-of-concept studies. Zhong and colleagues chose each surgeon’s average case duration of their three most recent radius-fracture procedures as a reference. Whether this reference is the most relevant to compete against may be debated. The strong advantage of the historical-mean reference is that it takes the individual surgeon into account. However, three cases are a limited sample, and it does not consider that the next procedure’s complexity may be different from the surgeon’s three most recent cases. For instance, senior surgeons may likely be assigned to the occasional complex fracture along with also handling simple cases. Senior surgeons’ average case duration may thus be higher and more variable compared to junior surgeons, who may predominantly be assigned straightforward cases, which have shorter and less variable case durations. This may be easy for a human scheduler to detect, but not available to the reference model, so the reported improvement caused by the machine learning model could be less in comparison with another simple reference model. In addition to averaging more than three cases, a reference model could perhaps have taken a fracture and dislocation classification into account. Importantly, this means that only a proxy of real-world performance improvement has been measured.

## Feature and target leakage

In cases where researchers are worried about a possible discriminatory bias in training data, for example by race, making the model blind to the race should solve the problem. But how is that done? If, say, some features of the notes correlate with race, the model may still learn a racial bias, allowing an unintended “feedback loop” that maintains a structural bias, if present in training data.

This is, to our knowledge, an unsolved problem, which must be mitigated by designing control loops based on domain expertise. For example, if individuals of African descent are systematically under-tested for diabetes, monitoring the testing frequency across races could flag the lower test-rate, which could be mitigated by a small amount of randomized testing [11].

Leakage can also occur for the outcome. During the training, the model simulates generating a prediction at a given time point. In the case of Zhong and colleagues, at the time of surgery scheduling. If the model gains access to information “from the future”, for example, a description of the surgery after it has been completed, it will perform better in training, but worse when implemented in real life, since that information will not be available. This is called target leakage or data leakage.

Zhong and colleagues are likely not vulnerable to this, since they use radiological descriptions, which should not be updated based on surgical results.

However, this is especially pertinent for text, since it is more frequently modified than e.g., lab results of blood samples. Researchers should know exactly how the underlying systems timestamp notes, not least if they are updated.

## Conclusion

Zhong and colleagues have made commendable strides in integrating both textual and structured data to predict surgical durations. Their work, whether explicitly or implicitly, addresses many of the aspects highlighted above. This consideration is paramount, not just for academic rigor, but more crucially, to ensure that prediction models can benefit patients and/or the health care system. They provide a proof-of-concept of their algorithm, and, more generally, they have identified an example where text analyses of clinical notes may become relevant within the anesthesia field: the planning of anesthetic/surgical procedures. Within anesthesia and intensive care, one could imagine other relevant LLM-based prediction models that made use of, e.g. echocardiographic notes and the notes during an ICU stay. We are likely to encounter more of such exciting studies in the near future.

## Data Availability

No datasets were generated or analysed during the current study.

## References

[1] Isensee F, Jaeger PF, Kohl SA, Petersen J, Maier-Hein KH (2021). nnU-Net: a self-configuring method for deep learning-based biomedical image segmentation. Nat Methods.

[2] Vistisen ST, Pollard TJ, Harris S, Lauritsen SM (2022). Artificial intelligence in the clinical setting: towards actual implementation of reliable outcome predictions. Eur J Anaesthesiol EJA.

[3] Danielsen AA, Fenger MHJ, Østergaard SD, Nielbo KL, Mors O (2019). Predicting mechanical restraint of psychiatric inpatients by applying machine learning on electronic health data. Acta Psychiatr Scand.

[4] Zhong W et al. Improving Case Duration Accuracy of Orthopedic Surgery Using Bidirectional Encoder Representations from Transformers (BERT) on Radiology Reports,., 2023. 10.1007/s10877-023-01070-w PMC10879219 37695448

[5] Devlin J, Chang M-W, Lee K, Toutanova K. BERT: Pre-training of Deep Bidirectional Transformers for Language Understanding, in *Proceedings of the* 2019 *Conference of the North American Chapter of the Association for Computational Linguistics: Human Language Technologies, Volume 1 (Long and Short Papers)*, Minneapolis, Minnesota: Association for Computational Linguistics, Jun. 2019, pp. 4171–4186. 10.18653/v1/N19-1423.

[6] Pennington J, Socher R, Manning CD. Glove: Global vectors for word representation, in *Proceedings of the* 2014 *conference on empirical methods in natural language processing (EMNLP)*, 2014, pp. 1532–1543.

[7] Schmidt RM. Recurrent neural networks (RNNs): a gentle introduction and overview. arXiv. Nov. 2019;23. 10.48550/arXiv.1912.05911.

[8] Vaswani A et al. Attention is all you need. Adv Neural Inf Process Syst, vol. 30, 2017.

[9] Brown TB et al. Jun., Language Models are Few-Shot Learners, *ArXiv200514165 Cs*, 2020, Accessed: Jun. 03, 2020. [Online]. Available: http://arxiv.org/abs/2005.14165.

[10] Huang K, Altosaar J, Ranganath R. ClinicalBERT: Modeling Clinical Notes and Predicting Hospital Readmission, arXiv, arXiv:1904.05342, Nov. 2020. 10.48550/arXiv.1904.05342.

[11] Sculley D et al. Hidden technical debt in machine learning systems, *Adv. Neural Inf. Process. Syst*, vol. 28, 2015, Accessed: Nov. 07, 2023. [Online]. Available: https://proceedings.neurips.cc/paper/5656-hidden-technical-debt-in-machine-learning-sy.

